# Prescription Opioid Use in Patients With and Without Systemic Lupus Erythematosus — Michigan Lupus Epidemiology and Surveillance Program, 2014–2015

**DOI:** 10.15585/mmwr.mm6838a2

**Published:** 2019-09-27

**Authors:** Emily C. Somers, Jiha Lee, Afton L. Hassett, Suzanna M. Zick, Siobán D. Harlow, Charles G. Helmick, Kamil E. Barbour, Caroline Gordon, Chad M. Brummett, Deeba Minhas, Amrita Padda, Lu Wang, W. Joseph McCune, Wendy Marder

**Affiliations:** ^1^Division of Rheumatology, Department of Internal Medicine, University of Michigan, Ann Arbor; ^2^Department of Environmental Health Sciences, University of Michigan, Ann Arbor; ^3^Department of Obstetrics and Gynecology, University of Michigan, Ann Arbor; ^4^Department of Anesthesiology, University of Michigan, Ann Arbor; ^5^Department of Family Medicine, University of Michigan, Ann Arbor; ^6^Department of Epidemiology, University of Michigan, Ann Arbor; ^7^Division of Population Health, National Center for Chronic Disease Prevention and Health Promotion, CDC; ^8^Rheumatology Research Group, Institute of Inflammation and Ageing, University of Birmingham, United Kingdom; ^9^Department of Biostatistics, University of Michigan, Ann Arbor.

Rheumatic diseases are a leading cause of chronic, noncancer pain. Systemic lupus erythematosus (SLE) is a chronic autoimmune rheumatic disease characterized by periodic flares that can result in irreversible target organ damage, including end-stage renal disease. Both intermittent and chronic musculoskeletal pain, as well as fibromyalgia (considered a centralized pain disorder due to dysregulation of pain processing in the central nervous system), are common in SLE. Opioids are generally not indicated for long-term management of musculoskeletal pain or centralized pain (fibromyalgia) because of lack of efficacy, safety issues ranging from adverse medical effects to overdose, and risk for addiction (*1*,*2*). In this study of 462 patients with SLE from the population-based Michigan Lupus Epidemiology and Surveillance (MILES) Cohort and 192 frequency-matched persons without SLE, nearly one third (31%) of SLE patients were using prescription opioids during the study period (2014–2015), compared with 8% of persons without SLE (p<0.001). Among the SLE patients using opioids, 97 (68%) were using them for >1 year, and 31 (22%) were concomitantly on two or more opioid medications. Among SLE patients, those using the emergency department (ED) were approximately twice as likely to use prescription opioids (odds ratio [OR] = 2.1; 95% confidence interval [CI] = 1.3–3.6; p = 0.004). In SLE, the combined contributions of underlying disease and adverse effects of immunosuppressive and glucocorticoid therapies already put patients at higher risk for some known adverse effects attributed to long-term opioid use. Addressing the widespread and long-term use of opioid therapy in SLE will require strategies aimed at preventing opioid initiation, tapering and discontinuation of opioids among patients who are not achieving treatment goals of reduced pain and increased function, and consideration of nonopioid pain management strategies.

The MILES Cohort includes patients with SLE from the precursor MILES Surveillance Registry (*3*), which comprised persons with incident and prevalent SLE during 2002–2005. Briefly, the Registry source population included residents of Wayne and Washtenaw counties in southeastern Michigan, a region encompassing Detroit and Ann Arbor (population approximately 2.4 million). All MILES Registry patients still living in or near this region during the 2014–2015 recruitment and enrollment period were eligible for inclusion in the Cohort. During this period, 192 persons who did not have SLE were recruited from a random sample of households in this region and frequency-matched to SLE patients by age, sex, race, and county of residence. Males were oversampled among this group to attain roughly equivalent numbers of males in both groups. Ethics approval was obtained from the Institutional Review Boards of the University of Michigan and Michigan Department of Health and Human Services; written, informed consent was obtained from all participants.

Data were collected through structured interviews conducted during February 2014–September 2015. Self-reported data included all prescription medications currently being taken and duration of use; long-term opioid use was defined as use for >1 year. ED use was considered one or more visits to an ED within the last 12 months. Patient-reported outcome measures included fibromyalgia* (*4*), pain and physical function,^†^ and depression and anxiety.^§^ Measures specific to patients included SLE duration, disease activity (*5*), and SLE-related damage resulting from disease or its treatment (*6*).

Chi-squared tests or independent two-sample t-tests were used for comparisons between groups. Two multivariable logistic regression models were used to evaluate factors associated with opioid use in the total study population (patients and nonpatients) and in SLE patients only. In multivariable analyses, potential confounders included the following a priori-specified covariates: age, sex, race, income, education, unemployment, health insurance type, patient-reported measures (ED use, fibromyalgia, pain, physical functioning, depression, anxiety; and, for SLE patients, illness duration, activity, and damage). Stata (version 15.1; StataCorp) was used for analyses.

The study population included 462 SLE patients and 192 nonpatients. Patients were more often female, unemployed, and more frequently reported ED use, fibromyalgia, pain, poor physical function, depression, and anxiety (Table 1). Overall, 143 (31%) patients and 15 (8%) nonpatients were currently using prescription opioids (p<0.01). Among persons currently using prescription opioids, median duration (3 years) and interquartile range (IQR) (first and third quartiles) were similar among patients and nonpatients (IQR = 1 and 5 years, and 2 and 3 years, respectively; p = 0.91). Among patients using prescription opioids, 97 (68%) were on therapy for >1 year (Table 2), and 31 (22%) were using two or more opioid medications concomitantly.

**TABLE 1:** Sociodemographic characteristics and patient-reported outcomes in patients with systemic lupus erythematosus (SLE) and frequency matched persons without SLE — Michigan Lupus Epidemiology and Surveillance (MILES) Program, 2014–2015

Characteristic	No. (%) or mean (SD)		P-value^†^
	SLE patients (n = 462)	Persons without SLE* (n = 192)	
**Age (yrs), mean (SD)**	53.3 (12.3)	53.6 (14.0)	0.78
**Sex^§^**			
Female	430 (93.1)	154 (80.2)	<0.01
Male	32 (6.9)	38 (19.8)	
**Race**			
White	233 (50.4)	107 (55.7)	0.47
Black	208 (45.0)	77 (40.1)	
Other/Unspecified	21 (4.5)	8 (4.2)	
**Income below U.S. median**	237 (51.3)	114 (59.4)	0.16
**Education level**			
Less than high school	41 (8.9)	16 (8.3)	0.76
High school diploma/GED	46 (10.0)	26 (13.5)	
Some college/Associate degree	201 (43.5)	83 (43.2)	
Bachelor’s degree	88 (19.1)	37 (19.3)	
Graduate/Professional degree	85 (18.4)	30 (15.6)	
**Unemployed^¶^**	176 (38.1)	55 (28.6)	0.02
**Insurance**			
Private/Other	206 (44.6)	96 (50.0)	0.20
Medicaid/Medicare	246 (53.2)	89 (46.4)	
Uninsured	10 (2.2)	7 (3.6)	
**Emergency department use**	213 (46.1)	56 (29.2)	<0.01
**Fibromyalgia**	190 (41.1)	25 (13.0)	<0.01
Pain score,**^,††^ mean (SD)	48.2 (27.0)	28.4 (27.8)	<0.01
Physical function score,**^,††^ mean (SD)	43.8 (30.2)	24.7 (27.9)	<0.01
**Depression score,^††^ mean (SD)**	51.8 (9.9)	49.0 (9.1)	<0.01
**Anxiety score,^††^ mean (SD)**	52.4 (10.1)	49.5 (9.2)	<0.01
**Prescription opioid use**			
Prescription opioid use (current)	143 (31.0)	15 (7.8)	<0.01
Duration opioid use (years; median, IQR)^§§^	3 (1, 5)	3 (2, 3)	0.91
Concomitant use of ≥2 opioids^§§^	31 (21.7)	0 (0)	0.04
**SLE-specific measures**			
SLE duration, years (median, IQR)	19.0 (14.0, 26.0)	NA	NA
SLE activity score (mean, SD)^††^	12.9 (8.1)	NA	NA
SLE damage score (median, IQR)^††^	5.0 (3.0, 8.0)	NA	NA

**Abbreviations:** GED = General Educational Development certificate; IQR = interquartile range (25th percentile, 75th percentile); NA = not applicable; SD = standard deviation.

* Persons without SLE were frequency matched by age, sex, race, and county.

**^†^** P-values calculated by Pearson's chi-squared test (categorical data) or two-sample t-test (continuous data).

^§^ Males were oversampled in persons without SLE to have roughly equivalent numbers of males in both groups.

^¶^ Considered unemployed if aged <65 years, not working over last 12 months, and not in school.

** For both the pain and physical function measures, scores were reversed from their original RAND Medical Outcomes Study 36-item Short-Form-Survey instrument values so that higher scores represent worse pain and physical functioning, respectively.

^††^ Higher score is worse.

^§§^ Among persons with current prescription opioid use.

**TABLE 2:** Characteristics of persons reporting current prescription opioid use — Michigan Lupus Epidemiology and Surveillance (MILES) Program, 2014–2015

Factor	No. (%) of prescription opioid users	
	SLE patients (n = 143)	Nonpatients (n = 15)
**Opioid use ≥1 year**		
Yes	97 (67.8)	12 (80.0)
No	46 (32.2)	3 (20.0)
**Age group (yrs)**		
18–44	36 (25.2)	1 (6.7)
45–64	82 (57.3)	10 (66.7)
≥65	25 (17.5)	4 (26.7)
**Sex**		
Female	135 (94.4)	12 (80.0)
Male	8 (5.6)	3 (20.0)
**Race**		
White	59 (41.3)	6 (40.0)
Black	76 (53.2)	8 (53.3)
Other/unknown	8 (5.6)	1 (6.7)
**Income**		
Income <U.S. median	96 (67.1)	13 (86.7)
Income ≥U.S. median	37 (25.9)	2 (13.3)
**Education**		
Less than high school	22 (15.4)	3 (20.0)
High school diploma/GED	17 (11.9)	5 (33.3)
Some college/Associate degree	76 (53.2)	4 (26.7)
Bachelor’s degree	14 (9.8)	3 (20.0)
Graduate/Professional degree	14 (9.8)	0 (0.0)
**Employment**		
Unemployed	81 (56.6)	9 (60.0)
Employed and/or in school	62 (43.4)	6 (40.0)
**Insurance**		
Private/Other	37 (25.9)	3 (20.0)
Medicaid/Medicare	105 (73.4)	12 (80.0)
None	1 (0.7)	0 (0.0)
**Patient-reported outcomes**		
**Emergency department use**		
Yes (in last 12 months)	96 (67.1)	9 (60.0)
No	45 (31.5)	6 (40.0)
**Fibromyalgia**		
Yes	89 (62.2)	10 (66.7)
No	54 (37.8)	5 (33.3)
Pain score*^,†^		
<70	60 (42.0)	6 (40.0)
≥70	83 (58.0)	9 (60.0)
Physical function score*^,†^		
<70	73 (51.1)	12 (80.0)
≥70	70 (49.0)	3 (20.0)
**Depression score^†,§^**		
<56.2	74 (51.8)	8 (53.3)
≥56.2	67 (46.9)	7 (46.7)
**Anxiety score^†,§^**		
<62.3	99 (69.2)	13 (86.7)
≥62.3	42 (29.4)	2 (13.3)
**SLE-specific measures**		
**SLE duration**		
<15 yrs	29 (20.3)	NA
≥15 yrs	113 (79.0)	NA
**SLE activity score^†^**		
SLAQ <12	37 (25.9)	NA
SLAQ ≥12	106 (74.1)	NA
**SLE damage score^†^**		
LDIQ <5	41 (28.7)	NA
LDIQ ≥5	102 (71.3)	NA

**Abbreviations:** GED = General Educational Development certificate; LDIQ = lupus damage index questionnaire; NA = not applicable; SLAQ = systemic lupus activity questionnaire; SLE = systemic lupus erythematosus.

* For both the pain and physical function measures, scores were reversed from their original RAND Medical Outcomes Study 36-item Short-Form-Survey instrument values so that higher scores represent worse pain and physical functioning, respectively. Cut-points reflect 2 standard deviations from the mean.

**^†^** Higher score is worse.

^§^ Patient-Reported Outcomes Measurement Information System depression and anxiety score cut-points were based on PROsetta Stone mapping to the Center for Epidemiologic Studies Depression and Generalized Anxiety Disorder 7-item scales, respectively.

Within the total study population, the odds of opioid use among SLE patients were 3 times higher than for nonpatients (OR = 3.4, 95% CI = 1.7–6.6; p <0.001) after accounting for demographic, psychosocial, and clinical factors (Table 3). In analyses of both the total study population and SLE patients only, prescription opioid use was twice as likely among persons who had at least one ED visit in the last 12 months (total population: OR = 2.2, 95% CI 1.4–3.6), SLE patients only: OR = 2.1, 95% CI = 1.3–3.6). Pain and reduced physical functioning were also significantly associated with opioid use when assessing the total population and SLE patients only; for each one standard deviation increase (worsening) in pain and physical function scores, the odds of opioid use were approximately 35% and 12% higher, respectively.

**TABLE 3:** Factors associated with prescription opioid use, based on separate multivariable logistic regression models* for the total study population and systemic lupus erythematosus (SLE) patients only — Michigan Lupus Epidemiology and Surveillance (MILES) Program, 2014–2015

Characteristic	Total study population (n = 654)			SLE patients only (n = 462)		
	Prescription opioid use prevalence	OR (95% CI)	p-value	Prescription opioid use prevalence	OR (95% CI)	p-value
**Patient status**						
Nonpatient	7.8%	referent	NA	NA	NA	NA
SLE	31.0%	3.36 (1.72–6.57)	<0.001	NA	NA	NA
**Age (yrs)**	NA	1.00 (0.98–1.02)	NS	NA	0.99 (0.96–1.01)	NS
**Sex**						
Male	15.7%	referent	NA	25.0%	referent	NA
Female	25.2%	0.80 (0.35–1.86)	NS	31.4%	0.78 (0.28–2.17)	NS
**Race**						
White	19.1%	referent	NA	25.3%	NA	NA
Black	29.5%	1.01 (0.62–1.66)	NS	36.5%	1.03 (0.60–1.76)	NS
Other/Unknown	31.0%	1.14 (0.35–3.70)	NS	38.1%	1.10 (0.30–4.07)	NS
**Income**						
>U.S. median	14.6%	referent	NA	18.7%	referent	NA
≤U.S. median	31.1%	1.21 (0.68–2.14)	NS	40.5%	1.14 (0.62–2.11)	NS
**Education (yrs)**	NA	0.93 (0.84–1.02)	NS	NA	0.92 (0.83–1.02)	NS
**Employment**						
Employed and/or in school	16.1%	referent	NA	21.7%	referent	NA
Unemployed	39.0%	1.32 (0.82–2.11)	NS	46.0%	1.21 (0.72–2.03)	NS
**Insurance**						
Private	13.3%	referent	NA	18.0%	referent	NA
Medicaid/Medicare	34.9%	1.45 (0.82–2.56)	NS	42.7%	1.60 (0.85–3.00)	NS
None	5.9%	0.39 (0.03–4.27)	NS	10.0%	0.43 (0.04–4.79)	NS
**Patient-reported outcomes**						
**Emergency department use**						
No visits	13.4%	referent	NA	18.3%	referent	NA
≥1 visit last 12 mos	39.0%	2.22 (1.39–3.55)	0.001	45.1%	2.14 (1.27–3.59)	0.004
**Fibromyalgia**						
No	13.4%	referent	NA	19.9%	referent	NA
Yes	46.1%	1.50 (0.89–2.54)	NS	46.8%	1.18 (0.64–2.16)	NS
**Pain score** ^†,§^	NA	1.35 (1.19–1.53)	<0.001	NA	1.36 (1.18–1.58)	<0.001
**Physical function score^†,§^**	NA	1.11 (1.00–1.24)	0.047	NA	1.13 (1.00–1.27)	0.042
**Depression score^§^**	NA	1.01 (0.97–1.05)	NS	NA	1.01 (0.97–1.05)	NS
**Anxiety score^§^**	NA	0.97 (0.94–1.01)	NS	NA	0.98 (0.94–1.02)	NS
**SLE-specific measures**						
SLE duration (years)	NA	NA	NA	NA	1.02 (0.99–1.05)	NS
Activity (SLAQ score) ^§^	NA	NA	NA	NA	1.01 (0.96–1.06)	NS
Damage (LDIQ score) ^§^	NA	NA	NA	NA	0.98 (0.92–1.05)	NS

**Abbreviations:** CI = confidence interval; LDIQ = lupus damage index questionnaire; NA = not applicable; NS = not significant; OR = odds ratio; SLAQ = systemic lupus activity questionnaire; SLE = systemic lupus erythematosus.

* Each multivariable model includes all listed factors (i.e., odds ratios are adjusted for all other variables listed in the table): SLE versus nonpatient status (for total population model), age, sex, race, income, education, employment, health insurance, emergency department use, fibromyalgia, pain score, physical function score, depression score, and anxiety score. The SLE patient only model also included SLE duration, SLE activity score, and SLE damage score.

^†^ For both the pain and physical function measures, scores were reversed from their original RAND Medical Outcomes Study 36-item Short-Form-Survey instrument values so that higher scores represent worse pain and physical functioning, respectively. For the regression models, the (reversed) pain and physical function scores were scaled by their standard deviations of 10; therefore, each unit change represents one standard deviation change.

^§^ Higher score is worse.

## Discussion

In this study documenting the extent of prescription opioid use in patients with SLE, nearly one third of SLE patients in a well-characterized cohort used prescription opioids during 2014–2015, compared with 8% of frequency-matched persons without SLE. Approximately 70% of the SLE patients taking prescription opioids were on opioid therapy for >1 year. The higher odds of prescription opioid use among patients persisted after accounting for several factors in multivariable models. ED use in the last 12 months was associated with opioid use in both the total population and among SLE patients.

The widespread and long-term use of prescription opioids among this cohort of patients with SLE was striking given lack of evidence regarding safety and efficacy of opioids for treating chronic pain associated with rheumatic disease (*1*,*7*). Particularly concerning is that some of the less appreciated medical risks associated with long-term opioid use, such as myocardial infarction, immunosuppression, and osteoporosis (*8*), are potentially compounded in persons with SLE, whose baseline risks for these comorbidities are elevated because of the underlying disease and adverse effects of immunosuppressive and glucocorticoid therapies. Further, recent preliminary data suggest that opioids are associated with increased mortality in lupus.^¶^

Whereas rheumatic diseases are a leading cause of chronic, noncancer pain (*7*), data on opioid use and associated outcomes in persons with rheumatic diseases are limited. One recent study of Medicare beneficiaries with rheumatoid arthritis estimated regular opioid use (three or more filled prescriptions or one or more filled 90-day prescription per calendar year) at approximately 40% (*9*). Together with the findings from this analysis, the prevalent use of opioids in at least two patient populations with rheumatic diseases supports the need for better understanding of prescribing patterns, risk factors associated with opioid initiation and long-term continuation, and pharmacoepidemiology related to adverse medical effects of opioids in these patients. Effective interventions in this population will need to couple tailored approaches for tapering and discontinuing opioids when indicated, along with prevention of opioid initiation and consideration of nonopioid pain management strategies.

Interventions to address opioid use in patients with rheumatic diseases will require a better understanding of pain management for patients with these complex, chronic conditions, whose sources of pain might be multiple, persistent, and severe, and which must be accurately diagnosed to be appropriately treated. Sources of SLE-related pain can include active inflammatory disease resulting in peripheral pain (e.g., arthritis), damage accrual attributable to the disease or its treatment (e.g., steroid-induced osteonecrosis or vertebral fractures), or centralized pain disorders, such as fibromyalgia, the prevalence of which is higher in patients with SLE than in the general population (*4*).

The findings in this report are subject to at least five limitations. First, prescription data were self-reported, which limited the ability to examine sources of opioid prescribing or dosing patterns in more detail and could have been subject to underreporting attributable to social desirability bias. Second, since the original SLE registry reflected the demographics of southeastern Michigan (which is predominantly black and white), Asians, Hispanics, and other groups were not well represented, and results might not be generalizable to the wider SLE population. Third, this report addresses prescription opioid use, but information on other potential opioid sources is unavailable. Fourth, these data reflect 2014–2015; trends in opioid prescribing and usage might have changed since then. Finally, the cross-sectional nature of this analysis precludes assessing temporal relationships for factors associated with prescription opioid use. Strengths of this study include starting from a population-based SLE registry, inclusion of relatively large numbers of well-defined patients with SLE, comparing to age-, sex-, race-, and county-matched persons without SLE, and use of validated patient-reported outcome measures to assess psychosocial and lupus-specific factors in relation to prescription opioid use.

In conclusion, during 2014–2015, one third of patients in a SLE cohort in southeastern Michigan were using prescription opioids, most for longer than 1 year. Given the risks for opioid therapy and the lack of pain efficacy data in SLE, it is important that clinicians managing SLE, including providers in EDs, be aware of the potential adverse effects of opioid therapy in these patients, consider nonopioid pain management strategies, and be familiar with guidance for opioid tapering or discontinuation when patients are not achieving treatment goals of reduced pain and increased function or when otherwise indicated (*2*).

**Summary**

**What is already known about this topic?**

Opioids are generally not indicated for pain in systemic lupus erythematosus (SLE) and other rheumatic diseases because of limited efficacy and risks for addiction and adverse health effects.

**What is added by this report?**

Nearly one third of patients with SLE in an established Michigan cohort used prescription opioids, with approximately two thirds of those using for >1 year. Emergency department use was associated with increased prescription opioid use.

**What are the implications for public health practice?**

Risks for long-term opioid therapy, including osteoporosis and cardiovascular disease, are concerning in SLE patients given their increased underlying risks for these comorbidities. Strategies for reducing opioid use are needed in rheumatic disease populations. Clinicians managing SLE, including providers in emergency departments, need to be aware of these risks and consider nonopioid pain management strategies.

## Footnotes

* Based on survey criteria for fibromyalgia.

† Based on RAND Medical Outcomes Study Short-Form-Survey instrument subscales, with reversed scores so that higher scores represent worse states. https://www.rand.org/health-care/surveys_tools/mos/36-item-short-form.html.

§ Based on National Institutes of Health Patient-Reported Outcomes Measurement Information System short forms 8b (depression) and 8a (emotional distress-anxiety). http://www.healthmeasures.net/explore-measurement-systems/promis.

¶ https://acrabstracts.org/abstract/opiod-use-and-death-in-chronic-pain-patients-with-systemic-lupus-erythematosis/.

## References

[1] Chou R, Turner JA, Devine EB, The effectiveness and risks of long-term opioid therapy for chronic pain: a systematic review for a National Institutes of Health Pathways to Prevention Workshop. Ann Intern Med 2015;162:276–86. 10.7326/M14-2559 25581257

[2] Dowell D, Haegerich TM, Chou R. CDC guideline for prescribing opioids for chronic pain—United States, 2016. MMWR Recomm Rep 2016;65(No. RR-1). 10.15585/mmwr.rr6501e1 26987082

[3] Somers EC, Marder W, Cagnoli P, Population-based incidence and prevalence of systemic lupus erythematosus: the Michigan Lupus Epidemiology and Surveillance Program. Arthritis Rheumatol 2014;66:369–78. 10.1002/art.38238 24504809 PMC4198147

[4] Wolfe F, Clauw DJ, Fitzcharles M-A, Fibromyalgia criteria and severity scales for clinical and epidemiological studies: a modification of the ACR Preliminary Diagnostic Criteria for Fibromyalgia. J Rheumatol 2011;38:1113–22. 10.3899/jrheum.100594 21285161

[5] Karlson EW, Daltroy LH, Rivest C, Validation of a Systemic Lupus Activity Questionnaire (SLAQ) for population studies. Lupus 2003;12:280–6. 10.1191/0961203303lu332oa 12729051

[6] Costenbader KH, Khamashta M, Ruiz-Garcia S, Development and initial validation of a self-assessed lupus organ damage instrument. Arthritis Care Res (Hoboken) 2010;62:559–68. 10.1002/acr.20193 20391512 PMC3179258

[7] Lang LJ, Pierer M, Stein C, Baerwald C. Opioids in rheumatic diseases. Ann N Y Acad Sci 2010;1193:111–6. 10.1111/j.1749-6632.2009.05343.x 20398015

[8] Baldini A, Von Korff M, Lin EH. A review of potential adverse effects of long-term opioid therapy: a practitioner’s guide. Prim Care Companion CNS Disord 2012;14:14. 10.4088/PCC.11m01326 23106029 PMC3466038

[9] Curtis JR, Xie F, Smith C, Changing trends in opioid use among patients with rheumatoid arthritis in the United States. Arthritis Rheumatol 2017;69:1733–40. 10.1002/art.40152 28635179

